# Application of Experimental Studies of Humidity and Temperature in the Time Domain to Determine the Physical Characteristics of a Perlite Concrete Partition

**DOI:** 10.3390/ma17194938

**Published:** 2024-10-09

**Authors:** Anna Szymczak-Graczyk, Gabriela Gajewska, Barbara Ksit, Ireneusz Laks, Wojciech Kostrzewski, Marek Urbaniak, Tomasz Pawlak

**Affiliations:** 1 Department of Construction and Geoengineering, Faculty of Environmental and Mechanical Engineering, Poznan University of Life Sciences, Piątkowska 94E, 60-649 Poznań, Poland; gabriela.gajewska43@gmail.com (G.G.); ireneusz.laks@up.poznan.pl (I.L.); wojciech.kostrzewski@up.poznan.pl (W.K.); marek.urbaniak@up.poznan.pl (M.U.); tomasz.pawlak1@up.poznan.pl (T.P.); 2 Institute of Building Engineering, Faculty of Civil and Transport Engineering, Poznan University of Technology, Piotrowo 5, 60-965 Poznań, Poland; barbara.ksit@put.poznan.pl

**Keywords:** perlite concrete, thermal conductivity coefficient, sustainable construction, homogeneous partitions, hygrothermal performance, humidity of perlite, heat transfer, single-layer wall

## Abstract

These days, the use of natural materials is required for sustainable and consequently plus-, zero- and low-energy construction. One of the main objectives of this research was to demonstrate that pelite concrete block masonry can be a structural and thermal insulation material. In order to determine the actual thermal insulation parameters of the building partition, in situ experimental research was carried out in real conditions, taking into account the temperature distribution at different heights of the partition. Empirical measurements were made at five designated heights of the partition with temperature and humidity parameters varying over time. The described experiment was intended to verify the technical parameters of perlite concrete in terms of its thermal insulation properties as a construction material used for vertical partitions. It was shown on the basis of the results obtained that the masonry made of perlite concrete blocks with dimensions of 24 × 24.5 × 37.5 cm laid on the mounting foam can be treated as a building element that meets both the structural and thermal insulation requirements of vertical single-layer partitions. However, it is important for the material to work in a dry environment, since, as shown, a wet perlite block has twice the thermal conductivity coefficient. The results of the measurements were confirmed, for they were known from the physics of buildings, the general principles of the formation of heat and the moisture flow in the analysed masonry of a perlite block. Illustrating this regularity is shown from the course of temperature and moisture in the walls. The proposed new building material is an alternative to walls with a layer of thermal insulation made of materials such as polystyrene or wool and fits into the concept of sustainable construction, acting against climate change, reducing building operating costs, improving living and working conditions as well as fulfilling international obligations regarding environmental goals.

## 1. Introduction

The growing ecological awareness of societies has contributed to the increased willingness to use naturally obtained materials that reduce energy losses in building walls. The increasing awareness of this fact may significantly determine investors’ decisions to ensure that a building does not negatively impact its surroundings, is low-emission and that its maintenance does not consume excessive energy. Analysing the global energy consumption of countries’ economies, it should be acknowledged that 40% of total energy goes to construction. What is worse, this energy comes almost entirely from non-renewable sources, which increases greenhouse gas emissions [1,2,3,4,5,6]. An important criterion for selecting a building material is its composition and, above all, the lack of harmful compounds that may be released into the environment. Every year, according to the World Health Organization (WHO), about 7 million people die prematurely due to air pollution. In order to reduce the existing problem, the WHO issued the document “European Environment—status and outlook 2020” describing renewed European activities to pursue sustainable development [7]. The document presents the fact that sustainable development must become a guiding principle, thereby being the basis for ambitious, coherent policies and actions undertaken throughout society [8]. Examples of a building’s negative impact on the environment include the use of harmful artificial building materials or excessive electricity consumption. One of the most important and user-first requirements is to ensure thermal comfort inside. This is because it is the most noticeable factor when a person enters a building [9]. Passive building systems, together with zero-energy and plus-energy buildings, are the most modern building solutions today. These buildings are characterised by the highest thermal comfort and extremely low thermal energy consumption (15 kWh/m^2^/year, which is, for example, less than 1.5 L of heating oil or 1.5 m^3^ of natural gas per m^2^/year, or 5 kWh of electricity using a heat pump) [10,11,12,13]. Following the standards given by the Passive House Institute, buildings are being designed to provide a high level of living comfort along with low energy consumption [14,15,16]. The use of natural materials is also one of the principles of bioclimatic design [17]. Nowadays, it is important to use the lowest possible energy consumption, as well as the associated low operating costs of facilities, hence the huge interest in the use of ecological materials [18,19,20,21,22]. Work [23] confirms that the use of lightweight aggregate concrete contributes to meeting building energy efficiency and sustainability goals.

An example of a material that meets the above assumption is perlite concrete, which is a composite of cement slurry, aggregate and perlite. Perlite is a natural material, a magma rock formed by the cooling of acidic volcanic lava (rhyolite glaze) in an aqueous environment. Perlite as a natural raw material is used 90% of the time in the construction industry, where it is currently used mainly for the production of thermal insulation materials, and 10% of the time in agriculture as a fertilizer [22,24,25,26,27,28,29,30,31,32,33,34]. In this article, a new product—a block made of perlite concrete—was studied in order to test its thermal and mechanical properties, with the authors’ assumption of launching a product that, in addition to having the characteristics of a thermal insulating material, will also be a sufficiently load-bearing material to meet the strength standards of single-layer walls. The authors’ idea is to create a new material for passive, zero-energy and plus-energy construction. Once material tests have been carried out and the procedural requirements have been met, the product will be available for sale.

Thermal conductivity as a physical property of building materials is one of the basic ones that determine the applicability of materials in the design of an external wall in sustainable construction. Porous materials are filled with air in various percentages. If pores are open or very large, air movement increases in the material. In case of moisture, pores fill with water, which increases the value of the thermal conductivity coefficient. For instance, in aerated concrete, it is assumed that an increase in the thermal conductivity coefficient λ is approx. 4.5% per 1% increase in moisture.

The value of the thermal conductivity coefficient is a property of a given material; it is a physical property of the body that describes the ability of a given substance to transmit its internal energy. Under the same conditions, more heat will be transmitted through a substance with greater thermal conductivity. The thermal conductivity coefficient is not a constant value and depends on factors such as body structure, pressure, temperature, density, moisture content or time from production [34,35,36,37,38,39,40,41,42,43,44].

Moisture content is a favourable factor of thermal conductivity occurring through building partitions. With an increase in the average air temperature, the thermal conductivity of porous, granular and fibrous materials also goes up. As studies [31,32,33,45] have shown, increases in the thermal conductivity coefficient are directly proportional to increases in air temperature; however, they are so insignificant that computational models assume constant temperatures over time. A steady state is assumed as the protection against moisture condensation inside the partition and on its surface and is based on the analysis of water vapour diffusion through the partition. The diffusion results from the difference in temperature and relative humidity between the air inside and outside of the partition.

In the literature, you can find works that study concrete blocks with the addition of other materials such as crushed brick, ground Styrofoam, ground car tires, etc. Work [46] concluded that using lightweight concrete in structural and non-structural building envelopes is a valuable method of reducing the amount of heat transfer and energy consumption owing to lightweight concrete compared to normal weight concrete. Article [47] showed that lightweight concrete blocks with the addition of crushed brick or Styrofoam can replace standard lightweight concrete blocks because of their desirable mechanical properties, as well as their better thermal properties. Article [48] is about the study of a composite concrete block consisting of polyurethane (PUR) foam sandwiched between two c-shaped concrete layers. The study included theoretical calculations of steady-state U-values, a numerical simulation using the finite element method (FEM) and in situ U-value monitoring using the heat-flow method (HFM). The tested blocks showed better thermal performance compared to that of a traditional block. The trend for building materials to contain recycled aggregate additives is already widespread. However, there is not much research and not many products approved for use with the addition of perlite. Paper [49] presents the current state of knowledge on the use of perlite as a construction additive. The construction industry insists on the use of perlite currently treated for construction purposes, so as to replace other aggregates with this ecological raw material. Unfortunately, there are still no clear and reproducible results that are the basis for launching the production of perlite concrete elements. The test results in [50] indicate that thermal conductivity is substantially improved with the use of perlite and a strong relationship between thermal conductivity and unit weight is obtained. The experimental investigation in [51] revealed that the usage of raw perlite in concrete production reduces the concrete unit mass, increases the concrete strength and, furthermore, the thermal conductivity of the concrete is improved. In order to verify the possibility of using perlite as a component of perlite concrete, which can be used to make blocks for external building partitions, a 1:1-scale test stand was built to test the physical properties of the prepared building partitions. The purpose of the work is to present a new product—a perlite concrete block, which will be used for building single-layer external walls. Its dual properties, i.e., load capacity and thermal insulation, will speed up the construction process, improve it, reduce costs and enable the use of a completely natural product, which is resistant to chemicals, non-toxic, harmless to health, biologically neutral and resistant to microorganisms and fungi.

## 2. Materials and Methods

A single-layer wall 48 cm thick made of perlite concrete blocks with dimensions of 24.0 × 24.5 × 37.5 cm was adopted for analysing. By using single-layer walls, inter-layer condensation is avoided, and thus the problems related to biodeterioration of building partitions are reduced to zero [52,53].

### 2.1. Description of Research Methodology

Heat transfer in solids, in this case in a single-layer wall, takes place by conduction and is described by Fourier’s law, which governs how heat diffuses through solid materials. According to it, the heat flux density is proportional to the temperature gradient measured along the heat-flow direction (1).

(1) $$\dot{Q}=-\lambda \cdot A\cdot gradT=-\lambda \cdot A\cdot \left(\frac{\partial T}{\partial x}+\frac{\partial T}{\partial y}+\frac{\partial T}{\partial z}\right)$$

where

$\dot{Q}$—heat flux;
λ—thermal conductivity of the material;
T—temperature;
A—A=h·b, where h—height of the wall, b—width of the wall.

For a one-dimensional problem, when heat moves only along one axis, the equation can be simplified to (2). The minus sign means that the direction of the heat flow is opposite to the direction of the axis x (heat is always transferred from the object with the higher temperature to the object with the lower temperature, so the negative value corresponds to the section dx measured along the direction of heat flow).

(2) $$\dot{Q}=-\lambda \cdot A\cdot \frac{\partial T}{\partial x}$$

After solving Equation (2) by separating variables (3) and integrating sides (4), we obtained (5)

(3) $$\dot{\frac{Q}{A}}dx=-\lambda dT$$

(4) $$\int_{x_{1}}^{x_{2}}\dot{\frac{Q}{A}}dx=-\int_{T_{1}}^{T_{2}}\lambda dT$$

where

T_1_—air temperature on the inner surface of the material;
T_2_—air temperature on the outer surface of the material;
x_2_–x_1_—thickness of the layer.

For the flat wall, where A is the quotient of height and width but does not depend on the thickness of the wall, (5) and (6) were obtained:

(5) $$\dot{\frac{Q}{A}}\cdot \left(x_{2}-x_{1}\right)=\lambda \left(T_{1}-T_{2}\right)$$

(6) $$\dot{Q}=\frac{\lambda A\left(T_{1}-T_{2}\right)}{\left(x_{2}-x_{1}\right)}$$

**Figure 1:**
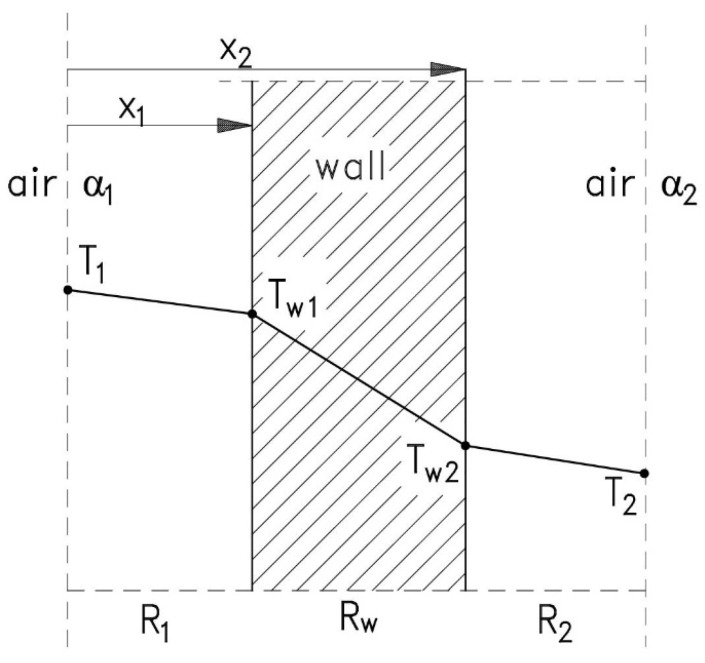
Diagram of heat transfer through a single-layer wall (based on [54]).

Heat transfer through a single-layer wall occurs from the place with the higher temperature to the place with the lower temperature (Figure 1).

According to Newton’s law, heat is first transferred from the air to the wall (7).

(7) $$\dot{Q}=A\cdot \alpha_{1}\cdot \left(T_{1}-T_{w1}\right)\to \frac{\dot{Q}}{A\cdot \alpha_{1}}=\left(T_{1}-T_{w1}\right)$$

Next, heat is conducted through the single-layer wall according to Fourier’s law (8).

(8) $$\dot{Q}=\frac{\lambda \cdot A\cdot \left(T_{w1}-T_{w2}\right)}{x_{2}-x_{1}}\to \frac{\dot{Q}}{\frac{\lambda \cdot A}{x_{2}-x_{1}}}=\left(T_{w1}-T_{w2}\right)$$

Finally, heat is transferred to the surroundings according to Newton’s law (9).

(9) $$\dot{Q}=A\cdot \alpha_{2}\cdot \left(T_{w2}-T_{2}\right)\to \frac{\dot{Q}}{A\cdot \alpha_{2}}=\left(T_{w2}-T_{2}\right)$$

By combining Formulas (7)–(9), Equation (10) was obtained, which can be written in terms of thermal resistances as (11) and (12) [54,55].

(10) $$\dot{Q}\left(\frac{1}{A\cdot \alpha_{1}}+\frac{1}{\frac{\lambda \cdot A}{x_{2}-x_{1}}}+\frac{1}{A\cdot \alpha_{2}}\right)=\left(T_{1}-T_{2}\right)$$

(11) $$\dot{Q}\left(R_{1}+R_{w}+R_{2}\right)=\left(T_{1}-T_{2}\right)$$

(12) $$\dot{Q}=\frac{\left(T_{1}-T_{2}\right)}{\left(R_{1}+R_{w}+R_{2}\right)}$$

where

$\dot{Q}$—heat flux;
λ—thermal conductivity of the material;
A—surface of the wall, A=h·b, where h—height of the wall, b—width of the wall;
T_1_—air temperature on the inner surface of the wall;
T_2_—air temperature on the outer surface of the wall;
x_2_ – x_1_—thickness of the layer;
T_w1_—air temperature on the inner surface of the wall;
T_w2_—air temperature on the outer surface of the wall;
R_1_—thermal resistance of air on the inner surface of the wall;
R_2_—thermal resistance of air on the outer surface of the wall;
R_w_—thermal resistance of the wall;
α_1_—heat transfer coefficient for the inner side;
α_2_—heat transfer coefficient for the outer side.

For thermal conductivity to depend on relative humidity, several factors must be taken into account, such as the initial (dry) thermal conductivity and how thermal conductivity changes with moisture content. The general formula for thermal conductivity including moisture can be written as (13) [54]:

(13) $$\lambda \left(w\right)=\lambda_{0}+k_{w}\cdot w$$

where

λ(w)—thermal conductivity of wet material;
λ_0_—thermal conductivity of dry material;
K_w_—coefficient of dependence of thermal conductivity on moisture;
W—moisture content of the material.

The dependence of thermal conductivity on moisture can also be described by Formula (14) [54]:

(14) $$\lambda \left(w\right)=\lambda_{0}\cdot (1+\alpha \cdot RH)$$

where

λ(w), λ_0_—as above;
α—coefficient of influence of RH relative humidity on thermal conductivity (dimensionless);
RH—relative humidity expressed as a fraction on a scale of 0 to 1 (e.g., 50% humidity is RH = 0.5).

The model describing the movement of moisture in building materials can be described by Formula (15) [54]:

(15) $$\lambda \left(w\right)=\lambda_{0}+\left(\lambda_{w}-\lambda_{a}\right)\cdot \theta_{w}$$

λ(w), λ_0_—as above;
λ_w_—thermal conductivity of water;
λ_a_—thermal conductivity of air;
θ_w_—the degree of filling the material with water, proportional to the moisture content (from 0 to 1).

A more complete numerical analysis for condensation is provided by Glaser’s method, which involves considering the boundary conditions for the model. The analysis is based on the rhythm of the annual cycle, which determines the increasing danger of internal condensation in winter. Only the annual moisture balance, taking into account the specific climatic conditions of the building’s location, can give information about the overall condition of the partition and the ability to neutralise winter adverse moisture [54]. In order to analyse the phenomena described earlier by empirical formulas, experimental studies were conducted throughout the year. On the basis of the data obtained and the calculation methods described in the next stage of the work, numerical calculations will be carried out.

### 2.2. Description of the Research

**Figure 2:**
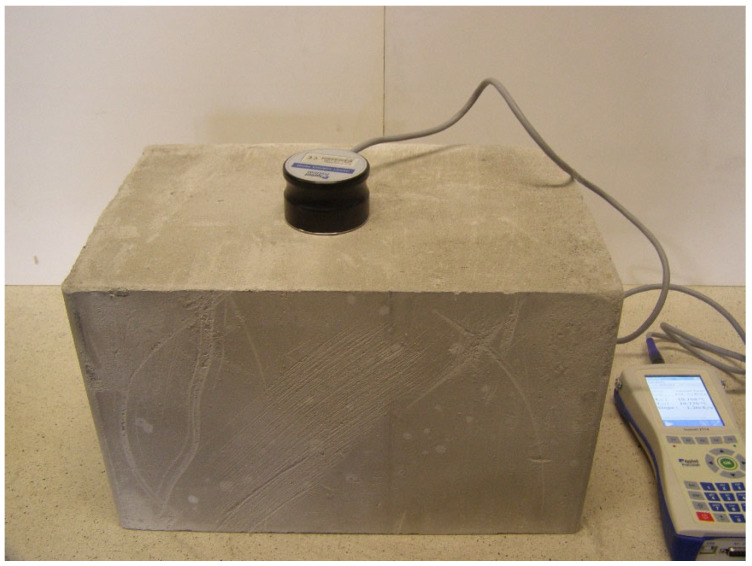
Perlite concrete block during the thermal conductivity test.

Tests were carried out on a prototype material—perlite concrete blocks. This is a product in the research phase, which will result in introducing it to the market (Figure 2). The blocks were made using Portland cement class 52.5 MPa and perlite. Bulk density of the blocks was 600 kg/m^3^; compressive strength was 0.9÷1.1 Mpa. Perlite concrete block is in the research phase, so physical parameters such as heat capacity and specific heat cannot be given at this time.

**Figure 3:**
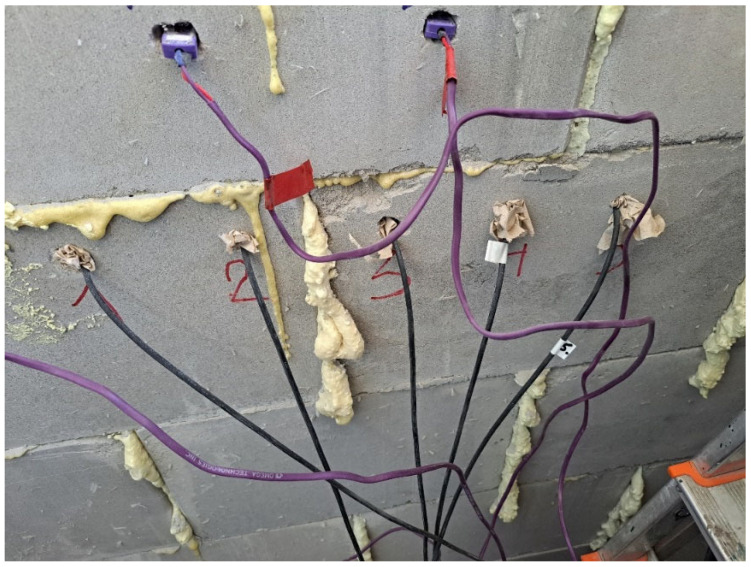
Arrangement of measurement points.

Tests consisted of recording temperature and humidity distributions in the partition of the tested object as well as inside and outside of the partition (Figure 3).

**Figure 4:**
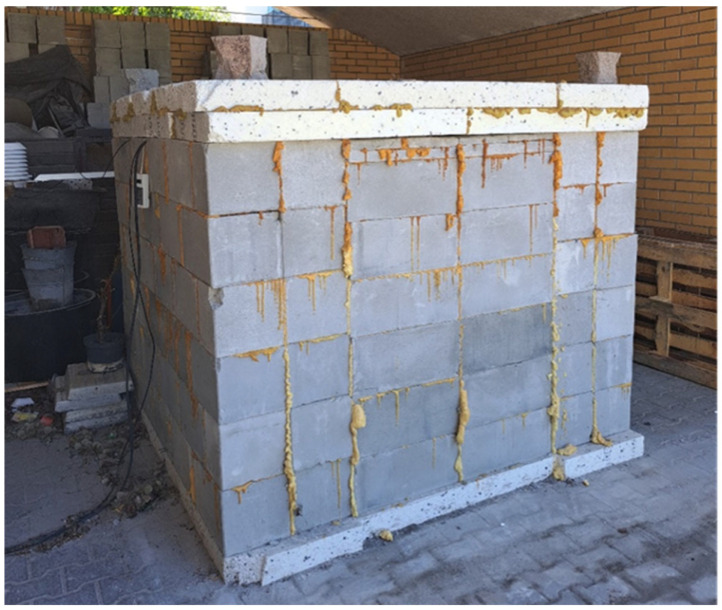
Research object.

The research object shown in Figure 4 had dimensions of 143.0 × 200.0 × 190.0 cm, with 48 cm thick walls. It was closed with a double layer of 100 mm thick EPS polystyrene. Five sensors were placed in the partition at depths of 46 cm, 36 cm, 26 cm, 16 cm and 5 cm. The object was equipped with a humidifier maintaining the set humidity, a heater maintaining the temperature and a DT85 recorder. From 23 November to 8 December, once the heater was turned on, inside temperature remained at approx. 46 °C; then, it was reduced to 36 °C. From 30 May, inside temperature was 26 °C.

Tests were carried out in situ on real partitions operating in the natural environment from 23 November 2022 to 13 June 2023. They involved measuring temperature and humidity at 5 points in the partition made of perlite concrete blocks, with temperature and humidity changing over time on the inner and outer side of the tested partition. Measuring points were evenly distributed at one height of the wall but at different depths so as to obtain accurate temperature and humidity curves. Temperature and humidity measurements were made every minute throughout the entire measurement period. This allowed for recording extreme values of temperature and humidity.

Thermal conductivity of a single block was determined on measurements made with an ISOMET 2114 device, AppliedPrecision (Bratislava, Slovakia), equipped with a surface probe. Air temperature and humidity measurements inside and outside the research object were made with HygroClip2 HC2A-S3, Rotronic sensors (New York, NY, USA), while humidity and temperature inside the walls were measured with T9602, Amphenol sensors. A DT85 ThermoFisher Scientific (Waltham, MA, USA) data logger was used to record data. Data were recorded every minute.

**Figure 5:**
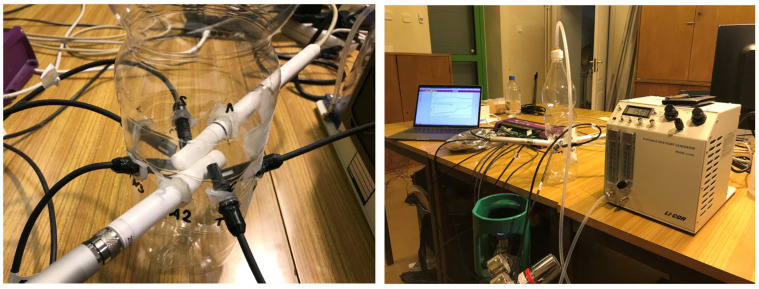
Test stand for calibrating relative humidity sensors.

Before installation in the research object, the sensors were calibrated and the differences between readings from the relative humidity sensors were verified (Figure 5). For this purpose, the sensors were placed in a bottle through which air with known water vapour content was passed. To achieve stable water vapour concentration conditions, a LI-610 dew-point generator, Licor, was used.

Due to minor differences between relative humidity readings from the sensors, a linear adjustment was used between the reading obtained from the dew-point generator (taken as the reference one) and readings obtained from thermo-hygrometers. Table 1 summarises the coefficients of the equation of a straight line used to equalise sensor readings.

In order to assess the transfer of water vapour through the tested wall, the actual vapour pressure (hPa) was calculated at each measurement point according to Formula (16):

(16) $$E=\frac{Rh}{100}E_{s}$$

where

Rh—relative humidity (%);
E_s_—saturation vapour pressure (hPa), computed according to the well-known Tetens’ Formula (17) in the following form [56,57]:

(17) $$E_{s}=6.113exp\left(\frac{17.2694T_{a}}{T_{a}+237.29}\right)$$

The risk of water vapour condensation in the wall material was assessed by using the water vapour pressure deficit (VPD) calculated as the difference between E_s_ and E.

## 3. Results

### 3.1. Dependence of the Thermal Conductivity Coefficient on Air Humidity

**Figure 6:**
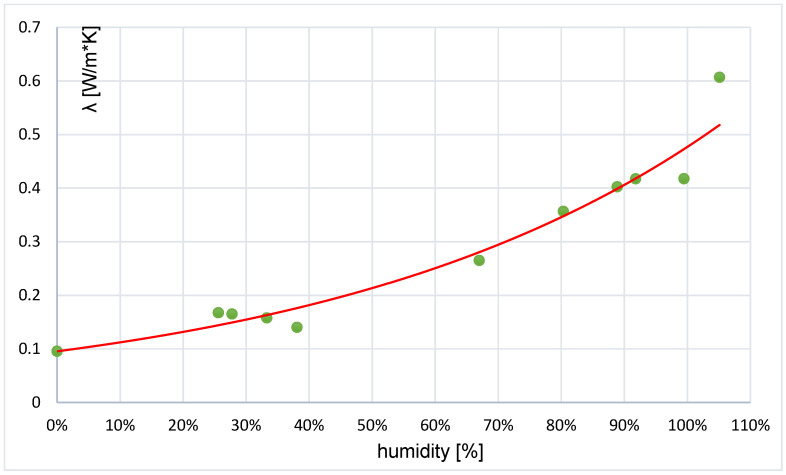
Influence of humidity on changes in the thermal conductivity coefficient λ of the perlite concrete block.

Tests of the thermal conductivity coefficient of a perlite concrete block in various humidity conditions were carried out using a portable ISOMET 2114 device equipped with a surface probe. The measurement results were used to create a graph depicting the influence of humidity on the value of the thermal conductivity coefficient. The graph curve has the form of a parabola and shows that the value of the thermal conductivity coefficient increases with increasing moisture content in the perlite concrete block (Figure 6). For the block in an air-dry state, the thermal conductivity coefficient λ is 0.0956 W/m·K, and for 50% humidity it is 0.215 W/m·K. This means that the value of the coefficient increases by more than twofold [31].

### 3.2. Humidity and Temperature Tests at the Thickness of the Partition

**Figure 7:**
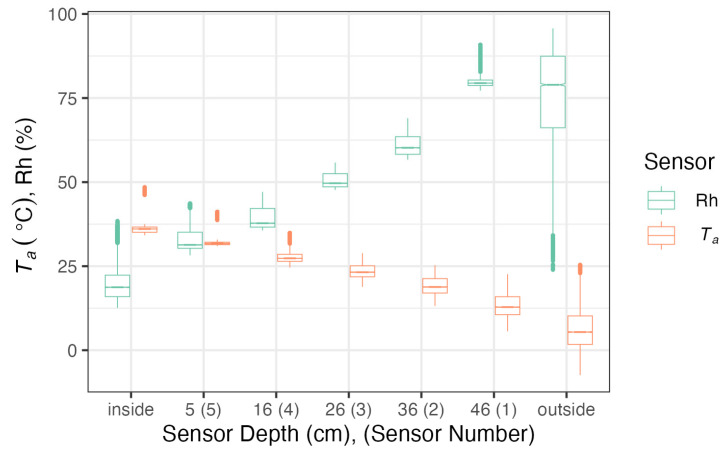
Boxplot of temperature and humidity distribution measured inside and outside the research object, and at various depths in the wall.

**Figure 8:**
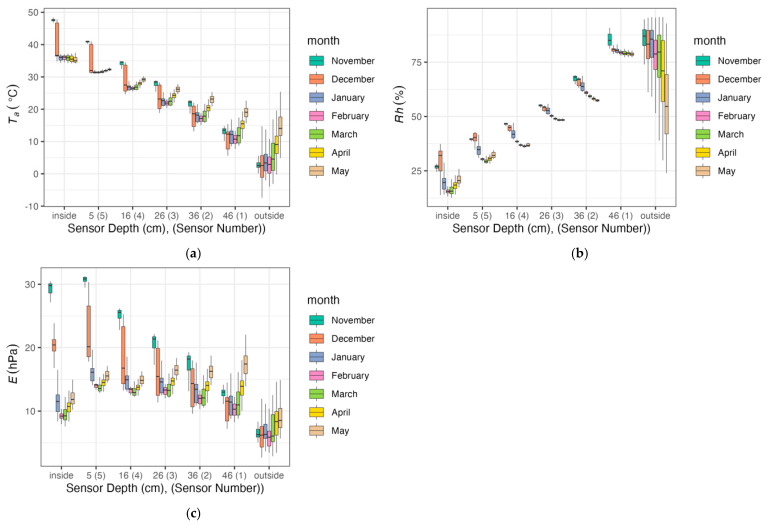
Thermo-hygroscopic parameters aggregated monthly: (**a**) temperature, (**b**) relative humidity, (**c**) actual water vapour pressure.

Humidity and temperature measurements were taken every minute during the measurement period, and their averaged values were recorded every half hour. Due to the large amount of measurement data from the entire measurement period, their average daily values were calculated. Adoption of the average measurement values is also justified by the fact that the change in the course of the humidity and temperature curves at the thickness of the partition is influenced primarily by the duration of given humidity or temperature. The collected measurement data provide a general and quite evident illustration of the thermal and hygroscopic conditions prevailing in the tested wall. Figure 7 shows the distribution of temperature and humidity measured inside and outside the research object, as well as at various depths in the wall, presented in the form of boxplots. As expected, the measured parameters show the greatest variability on the outer side. However, despite the use of a thermostat and constant air humidification inside the research object, variances in values at other measurement points are also visible. It seems that the data describing relative humidity of the air both in the surroundings (ambient) and inside, as well as inside the spaces in which the sensors were installed, do not carry an easy interpretation. This is due to the nature of relative humidity, which only informs about the degree of air saturation with moisture, not about the amount of water vapour. As a result, it is only possible to assess whether condensation may have occurred in the partition. In order to better understand the variability of thermo-hygroscopic conditions during the experiment, including the assessment of absolute moisture content and the assessment of moisture transfer through the partition, a number of parameters were calculated based on measurements to better reflect absolute moisture content in the air. Among them, actual water vapour pressure (E), saturated water vapour pressure (E_s_) and water vapour pressure deficit (VPD) were calculated. The results are presented in Figure 8 and divided into the subsequent months of the experiment, along with the results of temperature and humidity measurements.

Temperatures and humidities were measured inside (in the middle, 30 cm below the ceiling), and outside (at 200 cm height) the research object, and inside the wall at assigned depths. Boxplots indicate the spread of temperature and humidity (boxes show the first and third quartile, whiskers show the largest/smallest values but do not extend beyond 1.5 times of the interquartile range, and outliers are shown as separate points).

**Figure 9:**
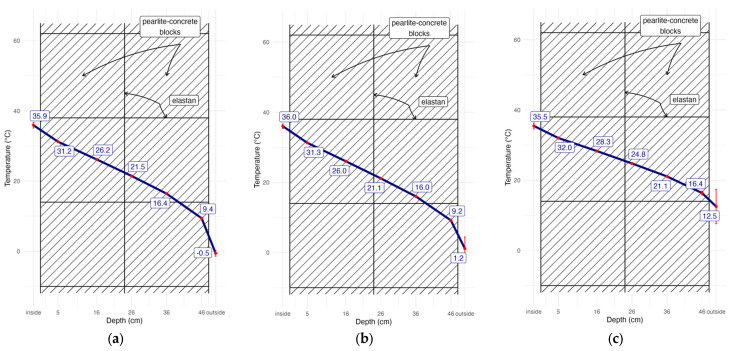
Temperature in the partition recorded: (**a**) on 20 January 2023, (**b**) on 1 March 2023, (**c**) on 1 May 2023 [32].

The obtained temperature data were used to draw graphs illustrating the temperature course at the thickness of the partition (Figure 9) on selected days.

### 3.3. Analysis of the Results

The graphs in Figure 7 and Figure 8a illustrate slight variations in temperature measured by all sensors. This was slightly influenced by both outside temperature and, to a small extent, by turning the thermostat-controlled heater on and off. During this period, the average daily air temperature on the inner side the partition ranged from +23.68 °C to +46.77 °C, and on the outer side, air temperature ranged from −4.58 °C to +17.58 °C. The highest temperatures were recorded by sensor no. 5 located closest to the inner side of the partition and were on average 5÷10 °C lower than the air temperature on the inner side of the partition. However, the lowest temperatures were recorded by sensor no. 1 located closest to the outer side of the partition. They were 5÷12 °C higher than the outside air temperature. Compared to temperature fluctuations, even larger fluctuations were recorded for relative air humidity. On the inner side of the partition, they ranged from 22.54% to 32.3%, and on the outer side from 36.03% to 95.4%. During this period, air humidity fluctuated greatly (Figure 8b). This is due to the fact that relative humidity strongly depends not only on the content of water vapour in the air, but also on temperature. This parameter describes the degree of air saturation with water vapour and does not describe the amount of moisture. It is also difficult to assess the directions of moisture transfer. Since water vapour, like any gas, spontaneously moves along the partial pressure gradient, the current value of water vapour pressure (E) was used to assess the directions of moisture transfer during measurements, which can also be used as a proxy for assessing moisture content in the material of the partition.

The values of the current water vapour pressure, both inside and outside the partition, suggest that for most of the duration of the experiment there were conditions in which the material of the partition tended to lose moisture in both directions (outside and inside). This is evidenced by slightly higher E values compared to those recorded both inside and outside the research object (Figure 8c). As outside temperature increased (April and May), an increase in E was observed in the outer layers of the partition. These values clearly exceeded the actual pressure of water vapour in the outdoor air. This proves that as the wall was heated from the outside, moisture content in the porous material began to evaporate more intensely, which resulted in an increase in water vapour pressure in the space where the sensor was placed. It can be assumed that in such conditions, the transfer of moisture from the interior of the building would be difficult because the water vapor pressure gradient inside the partition, although small, was reversed.

**Figure 10:**
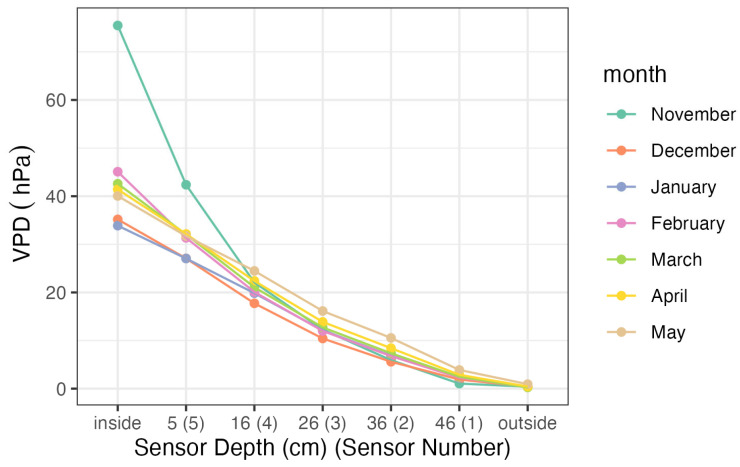
Minimum values of water vapour pressure deficiency at measurement points divided by months.

In order to investigate whether water vapour condensation could occur inside the partition at any time during the experiment, its vapour deficiency (VPD) was calculated. Conditions favouring condensation could appear during cold periods, and the possible accumulation of moisture and its condensation would be very undesirable also due to the deterioration of the thermal insulation properties of the tested material. The calculated minimum VPD values for individual sensors divided by months are shown in Figure 10 (condensation starts when VPD = 0). As it can be seen in the graph (Figure 10), the risk of condensation inside the material did not occur (even periodically) under the conditions of the experiment, which should be considered a valuable property of the tested material.

### 3.4. Statistical Analysis of the Results

**Figure 11:**
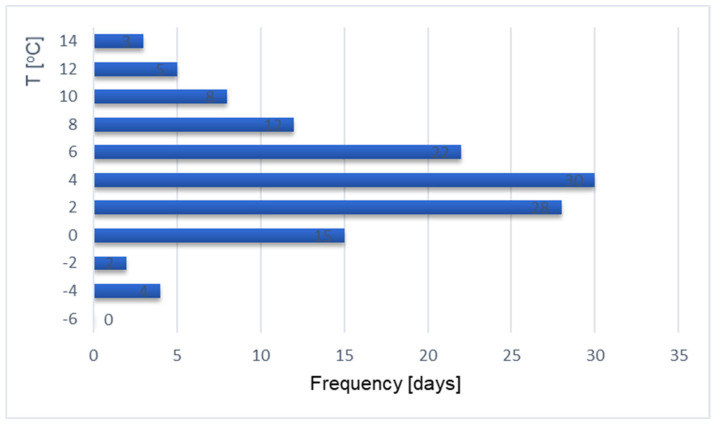
Frequency curve of temperature from 22 November 2022 to 31 March 2023.

The statistical analysis included temperature and humidity measurements in the winter period from 22 November 2022 to 31 March 2023. The collective characteristics of the external environment parameters influencing the partition are well described by the most frequently occurring temperature and humidity as well as the sum curves of the duration of these factors. Figure 11 shows a graph of the frequency of temperature occurrence in the winter period in question. The most frequent temperatures ranged from 4 to 6 °C.

**Figure 12:**
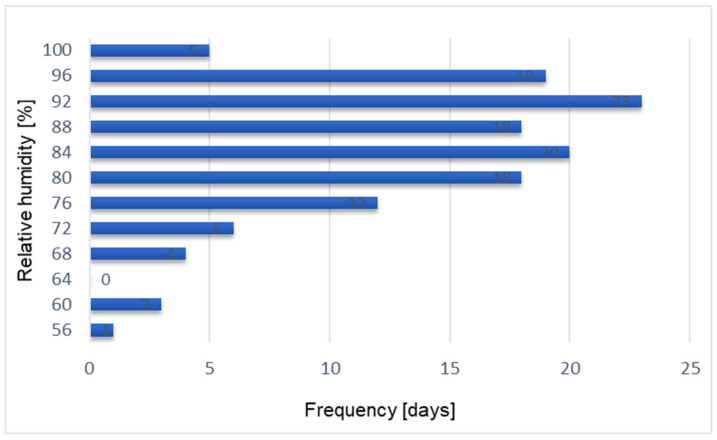
Frequency curve of relative humidity from 22 November 2022 to 31 March 2023.

The most frequent relative humidity values ranged from 92 to 96%, as shown in Figure 12.

**Figure 13:**
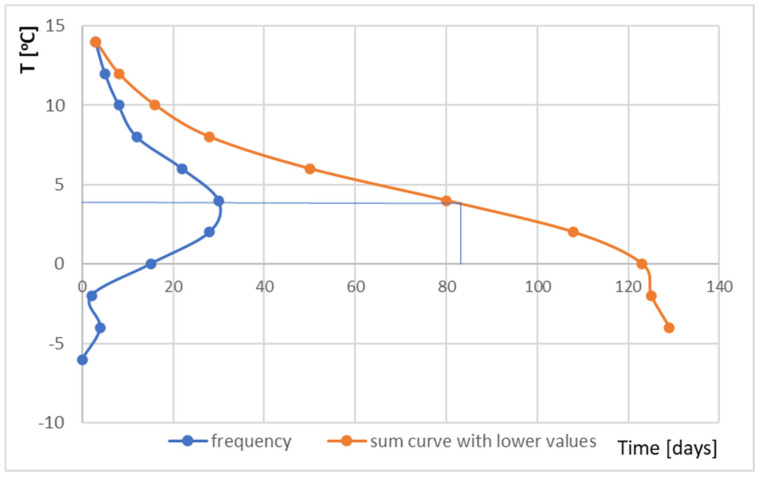
Frequency curve and the sum frequency curve incl. lower values for temperature from 22 November 2022 to 31 March 2023.

By plotting the sum curve with lower values (Figure 13), we can determine how many days the temperature was above or equal to the most frequently occurring temperature (in this case, it was 82 days) and how many days were below this temperature (47 days). For only 10 days, the temperature dropped below zero. The average temperature for this period is 3.44 °C and the median is 2.94 °C. It is worth noting that the distribution of temperature frequency for this period is normal. This finds confirmation in the Shapiro–Wilk test performed for this data set using RStudio [58]. The *p*-value is equal to 0.07022 for the significance level (α) equal to 0.05.

**Figure 14:**
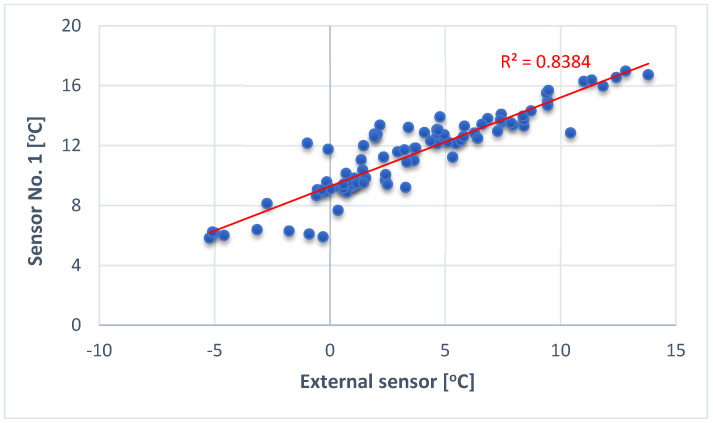
Linear correlation plot for data from sensor no. 1 and the external sensor.

Similar analyses were performed for the distribution of relative humidity. The number of days above or equal to the value that lasted the longest was 32, and the number of days below that value was 97. The average relative humidity for this period was 83.25%. The Shapiro–Wilk test [59] did not confirm the assumption of a normal distribution of relative humidity. The *p*-value is equal to 0.0002 and is lower than the assumed significance level (α) equal to 0.05. The median is 84.28%. A correlation analysis was also carried out between outside temperature and temperature recorded by the sensors installed in the partition. The correlation matrix calculated in RStudio showed that there is a very strong correlation only between the data from sensor no. 1 and outside temperature, in accordance with the ranges given in Table 2 [57]. The value of the correlation coefficient equals R2 = 0.8384, which is illustrated in Figure 14.

Sensor no. 1 was placed only 2 cm from the outer surface of the partition; hence, there may be a linear effect of external temperature, because thermal resistance of such a thin perlite layer is low. The correlations for the remaining sensors had values below 0.3, which, according to Table 2, are described as poor correlation. The statistical analyses carried out indicate that the examined period was characterised by a predominance of positive temperatures and high relative humidity. The maximum temperature gradient between the inside of the partition and the outer surface was 41.8 °C on 15 December 2022. The lack of correlation between external temperature and sensors no. 2,3,4,5 indicates strong insulating properties of the tested material.

**Figure 15:**
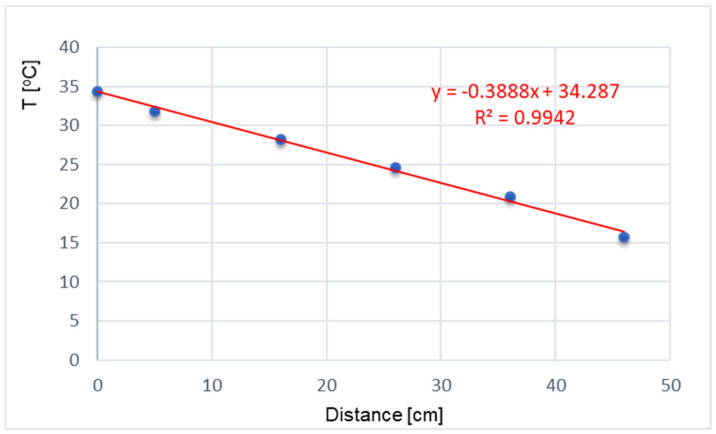
Temperature distribution and the linear regression curve in the partition for data recorded on 1 May 2023.

The course of temperature values recorded by the sensors inside the partition has a very strong linear correlation visible both in Figure 9 and in the graph presented in Figure 15. The correlation coefficient was calculated for the data presented in Figure 15 for inside temperature and for sensors no. 1 to 5. Outside temperature was omitted as an outlier. It is worth noting that the 2 cm layer of material between sensor no. 1 and the outer surface of the partition may not be as uniform as the remaining inner part of the partition, which may result from the action of external factors.

Similar analyses were carried out for a number of temperature distributions measured in different periods and their correlation coefficients were always above 0.98.

## 4. Discussion

This paper presents the results of experimental tests of a new building material introduced to the market. The perlite concrete block is intended to combine the prevailing trend of changing building habits and traditions in society, i.e., moving away from materials whose production consumes large amounts of energy in favour of partially or entirely sustainable materials. The authors’ idea is to develop a building material that will simultaneously meet strength and thermal insulation requirements.

In order to show the possibilities of using a single-layer wall made of perlite concrete blocks as an alternative to traditional solutions, popular among designers and investors, a comparison was made with other building partitions made of materials available on the market. The entire comparative analysis was referred to the standard [60] regarding the maximum value of the heat transfer coefficient for external walls of 0.20 W/(m^2^K) and is presented in Table 3. Explaining the values given in Table 3, it should be added that in the calculations carried out, the heat transfer resistance on the inner surface of the partition R_si_ = 0.13 m^2^K/W and the heat transfer resistance on the outer surface of the partition R_se_ = 0.04 m^2^K/W were taken into account according to [55]. For thermal materials with a water content of up to 10 kg/m^3^, the value of heat transfer coefficient λ = 0.04 W/mK was assumed for the calculation of a multilayer wall operating in an air-dry environment (dry conditions), while as the amount of water in the material increases, the heat transfer coefficient increases. The table assumes that for humid conditions, i.e., with a water content of 200 kg/m^3^, the coefficient value λ = 0.07 W/mK. Table 3 gives the U-factor for the element without taking into account corrections for mechanical fasteners piercing the insulation layer and insulation leaks (air voids). The corrections were not applied because the perlite concrete partition under consideration is single-material, and in the case of two-layer walls, the corrections would increase the U-factor. Also, no correction for air voids was taken into account because the calculation assumes a level of 0 leakage, according to Table F.1 of the standard [55]. The purpose of the table is to illustrate the variable U-value for different technologies.

Based on Table 3, it can be concluded that the single-layer wall made of perlite concrete blocks meets the requirements [60] and the value of the heat transfer coefficient of the partition is less than 0.20 W/(m^2^K). The advantages of the single-layer perlite concrete solution include material homogeneity, short construction time, greater durability of the external layer on which plaster is applied and the use of environmentally friendly material.

The new material—the perlite concrete block—requires further research and analysis. This work refers to the effect of humidity on the change in the thermal conductivity coefficient and provides the actual temperature distribution across the thickness of the partition. The results of the thermal conductivity coefficient obtained on the basis of experimental tests were compared with the studies available in the literature on the use of perlite [55,61,62].

For the block in an air-dry state, the thermal conductivity coefficient λ is 0.0956 W/mK, and for 50% humidity it is 0.215 W/mK. This means that the value of the coefficient increases more than twice [31] depending on humidity. Studies [60,61] provide the values of the thermal conductivity coefficient for the wall made of aerated concrete blocks at a temperature of 20 °C and humidity of 50%. According to [55], λ is in the range of 0.14–0.29 W/mK, whereas [47] provides the value of the λ coefficient in the range of 0.17–0.29W/mK. Therefore, the obtained results fall within the ranges of values reported in other works. The use of perlite as an addition to building materials such as mortars and plasters has been known for years, primarily due to its thermal insulation properties. According to [28], the thermal conductivity coefficient λ for pure expanded perlite is 0.045–0.059 W/mK, and for products containing it (mortars, plasters, concrete mixtures), it is approx. 0.08–0.25 W/mK. Study [29] presents the results of tests on the bending strength of elements made of mortar with the addition of perlite. Although their values indicate a reduction in strength, there was a noticeable increase in their thermal insulation properties. Currently, the main way of using perlite in construction is as a component of thermal insulation materials. Work [42] presents a new insulating product based on perlite as a variant to other well-known insulating products. Using numerical thermal conductivity design models, the authors of [42] made material formulations. The experimental sample was shaped into a rectangular plate. The results obtained from theoretical computational models were verified by performing an experiment in which the lowest value of λ for plates made with the addition of perlite was 0.0435 W/mK. The authors of article [62] performed a study to create a formulation of high-strength and also lightweight concrete with structural applications. Accordingly, in the concrete composite, traditional aggregate was replaced with perlite in the following proportions of 20%, 40%, 60%, 80% and 100%. Material properties were analysed for the compressive strength, flexural strength and thermal conductivity of six concrete mixtures. The concrete mix suggested by the authors with 100% of the expanded perlite aggregate instead of the traditional one and a unit weight of 1703 kg/m^3^ achieved decreasing the value of the thermal conductivity coefficient by approx. 62%, ranging from 1.81 to 0.69 W/mK and a compressive strength of 42 MPa after 28 days. This proves that perlite aggregate is an excellent component of structural concrete composites with enhanced thermal properties.

Study [33] presents test results showing that moisture content in the perlite concrete block has a very large impact on the value of the thermal conductivity coefficient. For instance, at humidity w = 30%, the thermal conductivity coefficient λ increases to 0.160 W/mK, i.e., approx. 70%. And at humidity w = 50%, it increases by more than 100%. Therefore, perlite concrete blocks in an air-dry state with sorption humidity should be used to construct walls. However, during transport, storage and construction, perlite concrete blocks require protection against rainfall.

## 5. Conclusions

Sustainable construction is becoming the dominant issue in modern societies. Therefore, modern building materials should be produced primarily in keeping with natural ingredients, and perlite is one such material. A further key advantage of perlite concrete blocks is that they can be used to create a single-layer wall that meets load capacity and thermal insulation requirements.

Based on the tests and analyses of the physical parameters of the blocks, conclusions can be made as follows:

- The value of the tested thermal conductivity coefficient depends on humidity. For the block in an air-dry state, it is 0.0956 W/mK, while for 50% humidity, it is 0.215 W/mK.
- The temperature distribution in the sensors illustrated in the average daily temperature graphs shows that the temperature recorded by sensor no. 5 is approx. 20 °C higher than the temperature recorded by sensor no. 1, i.e., the one closest to the outer side of the partition. It can be noticed that sensor no. 1 responded most to temperature changes occurring outside the partition. The impact of these changes on the remaining sensors was smaller the further away they were from the outer side of the partition. Since temperatures recorded by five sensors at individual points of the partition were different, the values of relative humidity were also different, which found confirmation in measurements. The differences in relative humidity values between sensor no. 5 and sensor no. 1 exceeded even 50%. The thermal conductivity coefficient depends on relative humidity. Since relative humidity was different across the thickness of the partition in the locations of the sensors, the value of the thermal conductivity coefficient was also different. Therefore, it seems justified to assume that in such a case the average value of the thermal conductivity coefficient should be determined.
- The graphs in Figure 7, Figure 8, Figure 9 and Figure 10 show that sensor no. 1 located closest to the outer side of the partition recorded higher relative humidity than air humidity outside the partition in some periods. This proves that perlite concrete blocks do not lose moisture quickly and their moisture persists for a long time. This leads to the conclusion that perlite concrete partitions require external protection against weather conditions. However, from the inner side, they are an excellent example of walls that have a positive effect on the internal microclimate.
- The heat transfer coefficient for the partition 48 cm thick made of perlite concrete blocks in an air-dry state is U = 0.192 W/m^2^K (Table 3); that is, it corresponds to the maximum value of this coefficient for external walls 0.20 W/(m^2^K) allowed by the Regulation of the Minister of Infrastructure on the technical conditions to be met by buildings and their location. If perlite concrete blocks are not air-dry, the requirement resulting from regulation will not be met.
- The relative humidity graphs drawn along the thickness of the partition indicate that the relative humidity inside the partition is higher than the relative humidity of the air of the inner side of the partition. This corresponds to the dependence of relative humidity on temperature.
- In order to better understand the behaviour of moisture inside the partition and to assess the directions of its transfer through the tested material, the actual pressure was calculated. The results indicated a tendency to release moisture from the tested material, even in winter. This suggests that when perlite concrete is used in walls, their thermal insulation properties may improve as a result of drying.
- During and under the experimental conditions, no conditions favouring condensation were observed inside the block, even in the most outer layer (2 cm). This allows for assuming that during typical use, i.e., in residential construction, perlite concrete will not get wet and, consequently, its thermal insulation properties will not deteriorate.

## Figures and Tables

**Table 1:** Coefficients of the linear relationship between readings from the dew-point generator and from thermo-hygrometers.

	Rh_in	Rh_out	Rh1	Rh2	Rh3	Rh4	Rh5
span	1.01	0.92	0.91	0.96	0.79	0.97	0.86
intercept	−0.48	3.68	2.61	0.54	10.84	0.59	7.54

**Table 2:** Values of the correlation coefficient [58].

Correlation Coefficient |r|	Importance
0–0.3	poor correlation
0.3–0.5	moderate correlation
0.5–0.7	strong correlation
0.7–1	very strong correlation

**Table 3:** Comparison of the heat transfer coefficient between the wall made of perlite concrete blocks and other traditional available solutions for wall partitions.

Partition Type No.	Layers in the Partition with the Thermal Conductivity Coefficient (1) Dry Conditions (2) Humid Conditions	Thickness of Individual Layer [cm]	Total Thickness of the Partition [cm]	Heat Transfer Coefficient of the Entire Partition Dry Conditions (1) [W/m^2^K]	Heat Transfer Coefficient of the Entire Partition Humid Conditions (2) [W/m^2^K]
1.	Perlite concrete block (discussed in this paper) λ_1_ = 0.0956 W/mK λ_2_ = 0.215 W/mK	48	48	0.192	0.416
2.	Block made of porous ceramics (ρ = 800 kg/m^3^) with plain mortar λ_1_ = 0.300 W/mK λ_2_ = no data	25	42	0.190	no data
	Thermal material λ_1_ = 0.040 W/mK λ_2_ = 0.070 W/mK	17			
3.	Solid ceramic brick (ρ = 1800 kg/m^3^) λ_1_ = 0.770 W/mK λ_2_ = 0.910 W/mK	25	44	0.191	0.317
	Thermal material λ_1_ = 0.040 W/mK λ_2_ = 0.070 W/mK	19			
4.	ALFA cinder block (ρ = 1800 kg/m^3^) λ_1_ = 0.700 W/mK λ_2_ = 0.750 W/mK	24	43	0.210	0.312
	Thermal material λ_1_ = 0.040 W/mK λ_2_ = 0.070 W/mK	19			
5.	600 aerated concrete (ρ = 600 kg/m^3^) with plain mortar λ_1_ = 0.300 W/mK λ_2_ = 0.350 W/mK	25	41	0.200	0.315
	Thermal material λ_1_ = 0.040 W/mK λ_2_ = 0.070 W/mK	16			
6.	Aerated concrete (ρ = 600 kg/m^3^) with heat-insulating mortar λ_1_ = 0.220 W/mK λ_2_ = 0.250 W/mK	25	40	0.198	0.302
	Thermal material λ_1_ = 0.040 W/mK λ_2_ = 0.070 W/mK	15			
7.	Solid silicate brick (ρ = 1900 kg/m^3^) λ_1_ = 0.900 W/mK λ_2_ = 1.00 W/mK	25	44	0.192	0.319
	Thermal material λ_1_ = 0.040 W/mK λ_2_ = 0.070 W/mK	19			
8.	Reinforced concrete λ_1_ = 1.700 W/mK λ_2_ = 1.800 W/mK	25	44	0.197	0.331
	Thermal material λ_1_ = 0.040 W/mK λ_2_ = 0.070 W/mK	19			

## Data Availability

The original contributions presented in the study are included in the article, further inquiries can be directed to the corresponding author.
